# Supplementary material for: Characteristics and risk factors for sibling incest
Source: PLoS One. 2024 Dec 3;19(12):e0314550. doi: 10.1371/journal.pone.0314550 (PMC11614286; doi:10.1371/journal.pone.0314550)
Supplement: S1 File — (PDF) [file pone.0314550.s001.pdf]

The full survey is available at
https://osf.io/6qmyk/?view_only=3575930005f94fee9b9fc624c6087b4f.

Characteristics of Sibling Incest

Participants answered questions about sexual contact with a maximum of two specific siblings; for each sibling that sexual contact was reported with, participants were asked which, if any, of the following reasons had caused or motivated the contact: curiosity, desire, romance, they were urged, they were forced, they were influenced, or they were playing a game. A dichotomous variable (yes/no) for each reason was computed.

Coercion

If sibling incest had occurred, participants indicated how often they or their sibling had consented to the sexual contact and the age of the involved sibling. The age of the sibling was used to calculate the age difference between the participant and their sibling. This research relied on self-report data, which could have biased reports of consent; it is possible that a participant reported that their sibling consented when their sibling had not. If non-consent, use of force, or an age gap larger than 5 years was present, the contact was coded as being coercive. If none of these elements were present, the sibling incest was coded as non-coercive. Within each instance of sibling incest that was reported, participants could also indicate who had initiated the contact. Thus, specific instances of coercive sibling incest were further categorized by whether the contact was ever initiated by the participant or by their sibling. If sibling incest was reported with two siblings, these three types of coercion (i.e., any coercion, participant-initiated coercion, and sibling-initiated coercion) were collapsed across the two siblings, such that each variable captured whether each type of coercion had ever occurred.

Cues of Relatedness

Proximity to siblings, especially when very young, was identified by Westermarck[1] as a key mechanism in the development of incest avoidance and gaps in early proximity are associated with higher levels of sibling incest.[2] Close proximity measured whether the participant had lived with their sibling and if so, at what age they began doing so – before the age of one, between ages two and six, or after age six. This ordinal scale was created for the present research.

Maternal-neonatal association – the degree to which mothers were involved with children when very young – has been proposed as a factor in the likelihood of incest.[2] To measure maternal-neonatal association, a modified version of the caregiving subscale from the Parental Engagement Scale was created.[3] The caregiving subscale, which assesses the amount of caregiving a parent does toward their child, was modified such that how much the participant witnesses these caregiving behaviours toward their sibling was assessed. The modified scale had 10 items which were rated on a 6-point Likert scale, which ranged from ‘not at all’ to ‘more than once a day’. The internal consistency of the modified scale was excellent (α = .94).

Participants were also asked how closely they resembled their siblings. This item was measured on a 7-point scale, ranging from ‘not very much,’ to ‘very much’. Higher scores indicated greater perceived physical resemblance between siblings.

Family Dysfunction

Sexual contact between family members may co-occur. Participants were asked whether they had ever had sexual contact with a caregiver, including, their parents, their stepparents, or another caregiver. A variable capturing whether any sexual contact with a parent or parental figure had ever occurred was then computed.

Juveniles who have sexually offended are much more likely to have childhood sexual abuse or physical abuse histories in particular.[4] The neglect subscale from the *Childhood Trauma Questionnaire* was used in this study.[5] This scale consisted of 5 items rated on a 5-point Likert scale (‘never’ to ‘very often’), assessing whether basic provisions, like food and clean clothes, were provided during childhood, and whether the participant had a responsible caregiver. Higher scores indicated greater neglect. Past research found that this subscale had low internal consistency ($\alpha = .58$),[6] however, in the present research the internal consistency was acceptable ($\alpha = .70$).

Another factor in explanations of sibling incest is family dysfunction, including parent conflict, parent problems, and intrafamilial violence.[7] This scale consisted of 6 items assessing how frequently the participant’s parents argued or fought (measured on a 5-point Likert scale ranging from ‘never’ to ‘very much’) and whether their parents had any substance use issues, had any mental illness, were unemployed, or had a criminal history (yes/no). The total score was computed by taking the mean of the items. This scale showed adequate internal consistency after one item was removed ($\alpha = .68$).

Family attitudes and practices about nudity at home are associated with sibling incest.[7] Participants rated how much nudity had been accepted in their family and how often they had seen either or both of their parents nude before they turned 18. Both items were rated on a 5-point Likert scale (‘not very much/ never’ to ‘very much/ very often’), such that higher scores reflected greater acceptance of nudity. The mean of these items was computed to create the scale, which showed excellent reliability ($\alpha = .86$).

Antisocial Tendencies

Illegal or problematic sexual behaviour has been consistently linked with evidence of childhood antisocial behaviour, which can be manifested in behaviour problems at home, school misconduct, or delinquency.[8] Four items were created to assess childhood antisociality, however, they yielded poor internal consistency ($\alpha = .61$). Two items were then dropped from the scale. The remaining two items, which assessed how often the participant had hit or how often they had shouted at others as a child, were summed to create the final score. Each item was scored on a 5-point Likert scale (response options ranged from ‘not very much/ never’ to ‘very much/ very

often'), such that higher scores indicated greater antisociality. This measure showed adequate internal consistency (α = .67).

In addition to antisocial behaviour, antisocial personality traits have been implicated in juvenile sexual offending.[9] To assess impulsivity, participants rated how much they enjoyed taking risks and how much they thought about a decision before making it on a 5-point Likert scale (the latter item was reverse-coded; response options ranged from 'not very much/ never' to 'very much/ very often'). Higher scores indicated greater impulsivity. This scale had low internal consistency (α = .34).

Childhood Popularity

Less popular children may be more likely to have sexual contact with siblings because they have fewer sexual opportunities with peers. Two items assessed childhood popularity. Participants were asked to rate how popular they had been in their peer group on a 5-point Likert scale ('not very much' to 'very much') and how many friends they had before they turned 18 on a 5-point scale (options included none, one, few, some, and many). These items were measured on different scales so they were turned into *Z*-scores before summing the items to create the final score. This scale had acceptable internal consistency (α = .75).

Atypical Sexuality

A sexual preference for prepubescent children (pedophilia) or pubescent children (hebephilia) is one of the most important factors in the onset and persistence of sexual offences against children.[10] A pedohebephilia index was computed to assess sexual attraction to children relative to sexual attraction toward adults. Participants reported how sexually attracted they were to adult women, girls, adult men, and boys on a 7-point Likert scale that ranged from 'not sexually attractive' to 'very sexually attractive'. The highest attraction score towards adults was subtracted from the highest score across the child categories. Positive scores indicated greater attraction to children (i.e., under the age of 15) than to adults.

This study used a shortened version of Friedrich et al.'s [11] *Child Sexual Behavior Inventory*. Only items describing self-directed sexual behaviours or behaviours directed toward adults were included. Five items from the scale measured the presence of sexual behaviours with other children. These items were removed because they could be assessing sexual contact with a sibling and thus would inflate any association between atypical childhood sexual behaviours and sibling incest. Past research has found that the original scale had acceptable internal consistency (α = .72).[11] The internal consistency of the shortened scale used in this research was excellent (α = .90).

Individuals showing evidence of hypersexuality – reflected in excessive sexual preoccupation, high sex drive, and compulsive sexual behaviour – are at greater risk of problematic sexual behaviour.[12] Participants rated how much they thought about sex on a daily basis, via a 7-point scale (ranging from 'rarely' to 'often'). Higher scores indicated greater hypersexuality.

Proximal Factors

Participants were asked to rate on a 7-point scale (‘not at all interesting’ to ‘extremely interesting’) how sexually interesting eight types of physical contact with their sibling were. The behaviours included kissing (both being the recipient and the kisser), sex (both being the recipient and the initiator), fondling, lying on top of one another, unintentional touching, and tripping and falling on one another. The scores for the items were averaged. Higher scores indicated greater sexual interest.

Participants were asked to rate on a 7-point scale (‘not at all disgusting’ to ‘extremely disgusting’) how disgusting eight types of physical contact with their sibling were (see above). The behaviours were the same as those included in the questions examining sexual interest in the participants' siblings. The scores for the items were averaged and higher scores indicated greater disgust.
