# Supplementary material for: Characteristics and risk factors for sibling incest
Source: PLoS One. 2024 Dec 3;19(12):e0314550. doi: 10.1371/journal.pone.0314550 (PMC11614286; doi:10.1371/journal.pone.0314550)
Supplement: S2 File — (PDF) [file pone.0314550.s002.pdf]

Data cleaning

Of the 3,003 participants who consented to participate in this study ($N_{\text{North America}}$ = 1,416; N_{Germany} = 1,587), 2,086 completed the survey ($N_{\text{North America}}$ = 1,293; N_{Germany} = 793). After data cleaning, 200 participants were removed because they failed a validity check (e.g., select 'no'; $N_{\text{North America}}$ = 147; N_{Germany} = 18) or because they completed the survey too quickly (less than 5 seconds per question; $N_{\text{North America}}$ = 35; completion time for the German sample were not recorded). Further, participants recruited via two of the recruitment platforms – sibling websites and the University of Ottawa's student recruitment portal, the Integrated System of Participation in Research – were removed because very few people had completed the survey from these sites (*n*= 23).

Data anlysis

Outliers (i.e., $Z > 3.29$) were detected for the following scales: antisocial parents, childhood neglect, childhood antisociality, atypical childhood sexual interests, and the pedohebephilia index. Outliers were also detected for some demographic variables: participant age, the total number of siblings, and the total number of opposite-sex siblings. It was possible to reduce outliers for the antisocial parents scale, the pedohebephilia index, the atypical childhood sexual behaviours scale, and the items assessing the total number of siblings and the number of opposite-sex siblings. In these cases, outliers were reduced such that the rank of the score was maintained. Each of the logistic regressions were computed with the unreduced and the reduced outliers, but this did not affect the results or their interpretation. As such, the results presented in the main paper reflect the raw data (i.e., outliers were not reduced).
