# Supplementary material for: Characteristics and risk factors for sibling incest
Source: PLoS One. 2024 Dec 3;19(12):e0314550. doi: 10.1371/journal.pone.0314550 (PMC11614286; doi:10.1371/journal.pone.0314550)
Supplement: S1 Table — (PDF) [file pone.0314550.s005.pdf]

S1 Table. Characteristics of sibling incest by age contact began.

	Frequency % (*n*/*N*)	Before age 12 % (*n*/*N*)	12 or older % (*n*/*N*)	*p*	$\chi^2(1)$
Any sibling incest		61.0 (139/228)	39.0 (89/228)		
Type of contact				.040	4.21
Exploratory[a]	70.2 (144/205)	75.2[c] (91/121)	61.5[d] (48/78)		
Consummatory[b]	29.8 (61/205)	24.8[c] (30/121)	38.5[d] (30/78)		
Reason for contact was curiosity				.726	.123
Yes	85.6 (190/222)	84.8[e] (112/132)	86.6[e] (71/82)		
No		15.2[e] (20/132)	13.4[e] (11/82)		
Reason for contact was desire				< .001	22.96
Yes	44.8 (99/221)	31.8[f] (42/132)	65.4[g] (53/81)		
No		68.2[f] (90/132)	34.6[g] (28/81)		
Reason for contact was romance				.001	10.73
Yes	19.7 (43/218)	13.0[h] (17/131)	31.6[i] (25/79)		
No		87.0[h] (114/131)	68.4[i] (54/79)		
Reason for contact was being urged				.218	1.52
Yes	22.0 (48/218)	25.6[j] (33/129)	18.3[j] (15/82)		
No		74.4[j] (96/129)	81.7[j] (67/82)		
Reason for contact was being forced				.172	1.86
Yes	15.3 (33/216)	18.1[k] (23/127)	11.1[k] (9/81)		
No		81.9[k] (104/127)	88.9[k] (72/81)		
Reason for contact was being influenced				.486	0.49
Yes	27.1 (59/218)	28.8[l] (38/132)	24.4[l] (19/78)		
No		71.2[l] (94/132)	75.6[l] (59/78)		
Reason for contact was playing a game				.004	8.12
Yes	73.2 (161/220)	80.3[m] (106/132)	62.5[n] (50/80)		
No		19.7[m] (26/132)	37.5[n] (30/80)		

Note. Matching superscripts within rows indicate that the values are not significantly different at $p < .05$. Superscripts that do not match within rows indicate the values are different at $p < .05$. [a]Exploratory contact defined as touching, consummatory defined as vaginal intercourse.[b]Rates of consummatory sexual contact increased as the degree of relatedness decreased. The frequency of

consummatory behaviours across different sibling types were: full siblings = 23.2% (36/155); half-siblings = 57.1% (20/35); and step-siblings = 60.0% (12/20).
