# Supplementary material for: Characteristics and risk factors for sibling incest
Source: PLoS One. 2024 Dec 3;19(12):e0314550. doi: 10.1371/journal.pone.0314550 (PMC11614286; doi:10.1371/journal.pone.0314550)
Supplement: S2 Table — Matching superscripts within rows indicate that the values are not significantly different at p < .05. Superscripts that do not match within rows indicate the values are different at p < .05. (PDF) [file pone.0314550.s006.pdf]

	Female % (n/N)	Male % (n/N)	p	$\chi^2(1)$
Reason for contact was curiosity			.020	5.40
Yes	81.1^{a} (107/132)	7.8^{b} (7/90)		
No	18.9^{a} (25/132)	92.2^{b} (83/90)		
Reason for contact was desire			< .001	30.83
Yes	29.5^{c} (39/132)	67.4^{d} (60/89)		
No	70.5^{c} (93/132)	32.6^{d} (29/89)		
Reason for contact was romance			< .001	14.19
Yes	11.5^{e} (15/131)	32.2^{f} (28/87)		
No	88.5^{e} (116/131)	67.8^{f} (59/87)		
Reason for contact was being urged			.055	3.67
Yes	26.3^{g} (35/133)	15.3^{g} (13/85)		
No	73.7^{g} (98/133)	84.7^{g} (72/85)		
Reason for contact was being forced			.098	2.74
Yes	18.6^{h} (24/129)	10.3^{h} (9/87)		
No	81.4^{h} (105/129)	89.7^{h} (78/87)		
Reason for contact was being influenced			.978	0.01
Yes	27.1^{i} (35/129)	27.0^{i} (24/89)		
No	72.9^{i} (94/129)	73.0^{i} (65/89)		
Reason for contact was playing a game			.852	0.04
Yes	72.7^{j} (96/132)	73.9^{j} (65/88)		
No	27.3^{j} (36/132)	26.1^{j} (23/88)		
