# Supplementary material for: Characteristics and risk factors for sibling incest
Source: PLoS One. 2024 Dec 3;19(12):e0314550. doi: 10.1371/journal.pone.0314550 (PMC11614286; doi:10.1371/journal.pone.0314550)
Supplement: S3 Table — Matching superscripts within rows indicate that the values are not significantly different at p < .05. Superscripts that do not match within rows indicate the values are different at p < .05. cParticipants reported whether they or their sibling had ever consented to sibling incest. If there was any evidence of non-consent (i.e., participants reported they or their sibling had not consented), ‘yes’ was scored for this variable. (PDF) [file pone.0314550.s007.pdf]

	Less than 5 years<br>% (*n*/*N*)	5 years or more<br>% (*n*/*N*)	*p*	$\chi^2(1)$
Reason for contact was being forced			.019	5.50
Yes	12.6[a]<br>(21/167)	27.5[b]<br>(11/40)		
No	87.4[a]<br>(146/167)	72.5[b]<br>(29/40)		
Any non-consent[c]			.190	1.72
Yes	17.3[d]<br>(30/173)	26.2[d]<br>(11/42)		
No	82.7[d]<br>(143/173)	73.8[d]<br>(31/42)		
