# Supplementary material for: Characteristics and risk factors for sibling incest
Source: PLoS One. 2024 Dec 3;19(12):e0314550. doi: 10.1371/journal.pone.0314550 (PMC11614286; doi:10.1371/journal.pone.0314550)
Supplement: S4 Table — Values represent standardized coefficients. (PDF) [file pone.0314550.s008.pdf]

	Standardized estimate (SE)	*p*
Sexual interest in a sibling		
Constant proximity (a)	-0.154 (0.02)	< .001
Sibling incest		
Sexual interest in a sibling (b)	0.266 (0.02)	< .001
Constant proximity (c)	-0.027 (0.02)	.242
