# Supplementary material for: Characteristics and risk factors for sibling incest
Source: PLoS One. 2024 Dec 3;19(12):e0314550. doi: 10.1371/journal.pone.0314550 (PMC11614286; doi:10.1371/journal.pone.0314550)
Supplement: S5 Table — Coercion occurred if, in an instance of sibling incest, either sibling did not consent (i.e., the participant reported that they or their sibling had not consented), there was an age gap of more than 5 years between the siblings, or force was used. Matching superscripts within rows indicate that the values are not significantly different at p < .05. Superscripts that do not match within rows indicate the values are different at p < .05. (PDF) [file pone.0314550.s009.pdf]

	Frequency<br>%<br>(*n*/*N*)	Female<br>%<br>(*n*/*N*)	Male<br>%<br>(*n*/*N*)	*p*	$\chi^2(1)$
Any coercion[a]				.009	6.89
Yes	49.0<br>(117/239)	56.0[a]<br>(79/141)	38.8[b]<br>(38/98)		
No		44.0[a]<br>(62/141)	61.2[b]<br>(60/98)		
Sibling-perpetrated coercion				< .001	16.19
Yes	24.7<br>(59/239)	34.0[c]<br>(48/141)	11.2[d]<br>(11/98)		
No		66.0[c]<br>(93/141)	88.8[d]<br>(87/98)		
Participant-perpetrated coercion				.593	0.29
Yes	21.8<br>(52/239)	20.6[e]<br>(29/141)	23.5[e]<br>(23/98)		
No		79.4[e]<br>(112/141)	76.5[e]<br>(75/98)		
