# Supplementary material for: Characteristics and risk factors for sibling incest
Source: PLoS One. 2024 Dec 3;19(12):e0314550. doi: 10.1371/journal.pone.0314550 (PMC11614286; doi:10.1371/journal.pone.0314550)
Supplement: S6 Table — AOR = adjusted odds ratios; odds ratios adjusted for other variables in the model. Sibling incest was defined as coercive if there was no consent, if force or threat were used to achieve the contact, or if there was an age difference of 5 years or more between siblings. Non-coercive sibling incest was consensual, did not involve force or threats, and occurred between siblings with less than a 5-year age gap. R2 ModelCoercive = .41 (n = 1,190/1,863; number of incidents = 49); R2 ModelNon-coercive = .30 (n = 1,213/1,863; number of incidents = 86). Sample sizes for coercive ORs ranged from 1,376 to 1,659 (mdn = 1,646) and for non-coercive ranged from 1,401 to 1,696 (mdn = 1,683). (PDF) [file pone.0314550.s010.pdf]

	Participant-instigated coercive				Participant-instigated non-coercive			
	OR [95% CI]	*p*	AOR [95% CI]	*p*	OR [95% CI]	*p*	AOR [95% CI]	*p*
Biological sex (male)	2.01 [1.14, 3.55]	.017	0.99 [0.36, 2.72]	.981	2.90 [1.87, 4.51]	< .001	1.24 [0.60, 2.56]	.554
**Cues of Relatedness**								
Close proximity	0.76 [0.60, 0.96]	.023	1.23 [0.78, 1.95]	.367	0.98 [0.79, 1.23]	.866	1.16 [0.81, 1.65]	.426
Maternal-neonatal association	1.04 [0.85, 1.27]	.727	1.04 [0.75, 1.45]	.809	1.00 [0.86, 1.17]	.976	1.04 [0.82, 1.32]	.743
Physical resemblance	0.99 [0.84, 1.17]	.918	0.97 [0.76, 1.25]	.830	0.88 [0.77, 0.99]	.037	0.93 [0.77, 1.12]	.437
**Family Dysfunction**								
Sexual abuse by a parent (yes)	18.08 [9.45, 34.60]	< .001	3.24 [0.96, 10.86]	.057	6.65 [3.56, 12.43]	< .001	1.62 [0.50, 5.25]	.420
Childhood neglect	3.16 [2.26, 4.41]	< .001	1.10 [0.48, 2.53]	.819	1.94 [1.45, 2.60]	< .001	1.12 [0.56, 2.23]	.749
Antisocial parents	2.78 [1.87, 4.14]	< .001	1.28 [0.56, 2.94]	.556	1.27 [0.85, 1.91]	.243	0.74 [0.37, 1.51]	.410
Positive family attitudes toward nudity	1.31 [1.04, 1.63]	.020	1.02 [0.70, 1.49]	.900	1.65 [1.39, 1.96]	< .001	1.41 [1.09, 1.83]	.009
**Antisocial Tendencies**								
Childhood antisociality	1.27 [1.10, 1.47]	.001	0.90 [0.70, 1.16]	.409	1.19 [1.06, 1.34]	.003	0.93 [0.77, 1.13]	.445
Childhood impulsivity	1.92 [1.36, 2.71]	< .001	1.85 [1.05, 3.25]	.033	1.81 [1.39, 2.37]	< .001	1.40 [0.93, 2.10]	.112
Childhood popularity	0.87 [0.66, 1.14]	.310	0.77 [0.51, 1.16]	.207	1.19 [0.96, 1.47]	.115	0.83 [0.61, 1.13]	.226
**Atypical Sexuality**								
Sexual interest in children	1.58 [1.40, 1.79]	< .001	1.30 [1.08, 1.57]	.006	1.20 [1.09, 1.32]	< .001	1.06 [0.91, 1.23]	.481
Atypical childhood sexual behaviours	1.22 [1.17, 1.27]	< .001	1.17 [1.08, 1.26]	< .001	1.20 [1.16, 1.24]	< .001	1.11 [1.05, 1.18]	< .001
Hypersexuality	1.18 [1.00, 1.39]	.048	0.87 [0.66, 1.14]	.316	1.64 [1.42, 1.89]	< .001	1.12 [0.91, 1.37]	.289
**Proximal Factors**								
Disgust toward sibling incest	0.54 [0.46, 0.63]	< .001	0.71 [0.53, 0.95]	.020	0.51 [0.45, 0.58]	< .001	0.66 [0.54, 0.82]	< .001
Sexual interest in sibling	1.57 [1.39, 1.77]	< .001	1.17 [0.90, 1.52]	.232	1.60 [1.45, 1.76]	< .001	1.18 [0.97, 1.44]	.095
