# Supplementary material for: Characteristics and risk factors for sibling incest
Source: PLoS One. 2024 Dec 3;19(12):e0314550. doi: 10.1371/journal.pone.0314550 (PMC11614286; doi:10.1371/journal.pone.0314550)
Supplement: S7 Table — a p < .001, bp < .01. nProximity between 1,818 and 1,828; nMaternal-neonatal between 1,764 and 1,772; nResemblance between 1,838 and 1,848. (PDF) [file pone.0314550.s011.pdf]

Cues of relatedness	Proximal Factors	
	Sexual interest in sibling<br>*r* [95% CI]	Disgust toward sibling incest<br>*r* [95% CI]
Close proximity	- .15[a] [-.20, -.11]	.06 [b] [.02, .11]
Maternal-neonatal association	- .03 [-.08, .02]	.01 [-.03, .06]
Physical resemblance	- .02 [-.07, .03]	- .03 [-.07, .02]
