# Supplementary figures and images for: Characteristics and risk factors for sibling incest
Source: PLoS One. 2024 Dec 3;19(12):e0314550. doi: 10.1371/journal.pone.0314550 (PMC11614286; doi:10.1371/journal.pone.0314550)

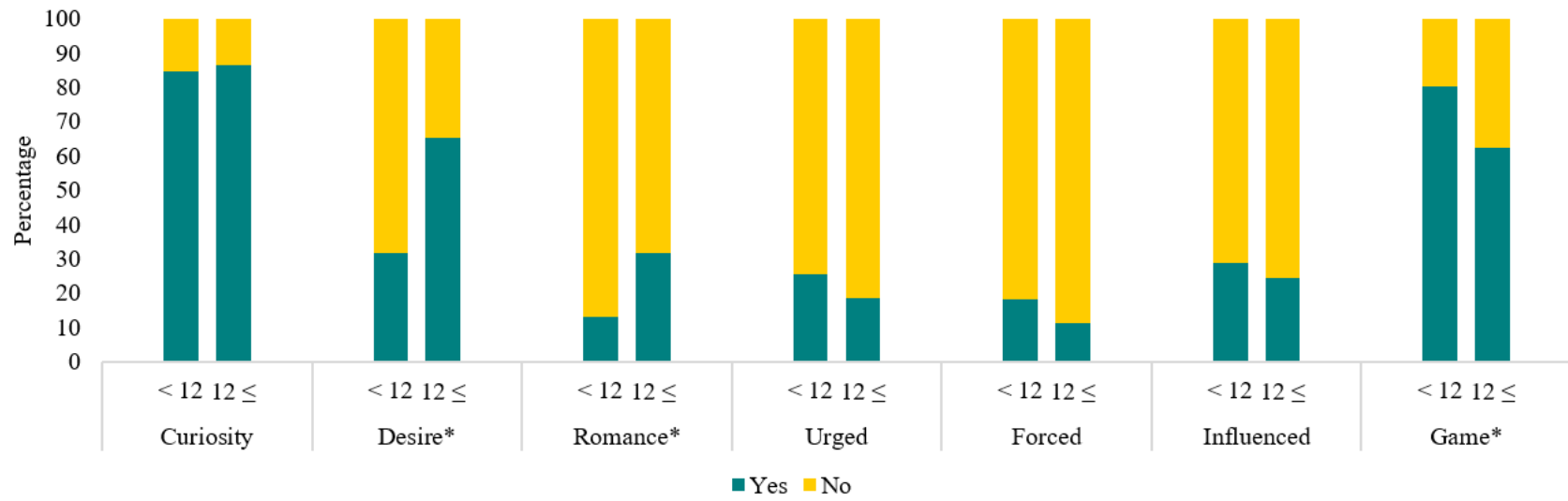

Supplement: S1 Fig — Asterisks denote significant group differences, p < .05, all n between 123 and 126. (PDF) [file pone.0314550.s012.pdf]

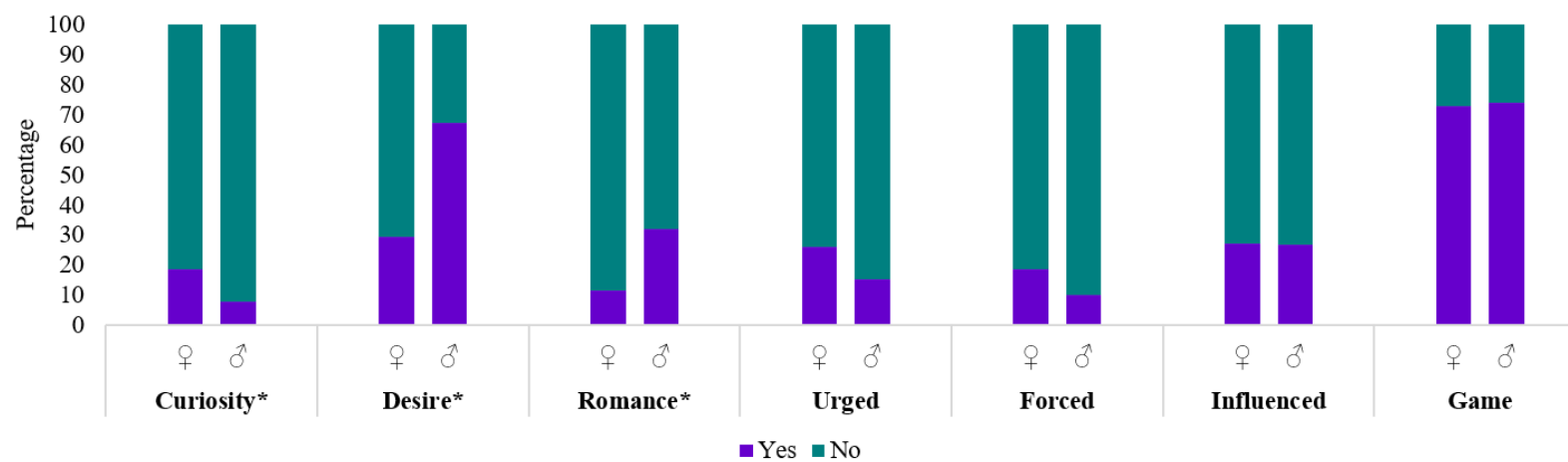

Supplement: S2 Fig — Asterisks denote significant group differences, p < .05, all n between 216 and 222. (PDF) [file pone.0314550.s013.pdf]

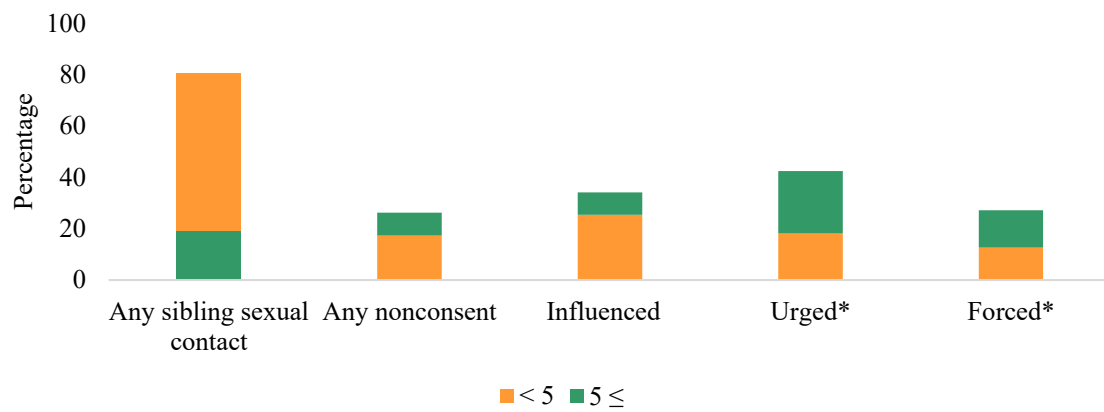

Supplement: S3 Fig — Asterisks denote significant group differences, p < .05, all n between 207 and 227. (PDF) [file pone.0314550.s014.pdf]
